# A case report and literature review on a large MDM2 negative retroperitoneal/psoas muscle well-differentiated liposarcoma mimicking intramuscular myxoma

**DOI:** 10.1016/j.radcr.2025.05.094

**Published:** 2025-06-26

**Authors:** Victoria Xie, Guiliang Yu, Amanda Wiggett, Justin Rivard, Miao Lu, Yi Yan

**Affiliations:** a Rady Faculty of Health Sciences, Interdisciplinary Health Program, University of Manitoba, Winnipeg, Manitoba, Canada; b Department of Urology, Shenzhen Baoan Hospital, The Second Affiliated Hospital of Shenzhen University, Shenzhen, China; c Department of Pathology, Max Rady College of Medicine, University of Manitoba, Health Sciences Centre, Winnipeg, Manitoba, Canada; d Department of Surgery, Max Rady College of Medicine, University of Manitoba, Winnipeg, Manitoba, Canada; e Department of Diagnostic Radiology, Max Rady College of Medicine, University of Manitoba, Winnipeg, Manitoba, Canada

**Keywords:** Well-differentiated liposarcoma, Intramuscular myxoma, Myxoid neoplasm, Core biopsy, MDM2 gene

## Abstract

Well-differentiated liposarcoma (WDLPS) typically presents as a large soft tissue mass in the retroperitoneal region. Amplification of the MDM2 gene is considered the gold standard for pathologic diagnosis. We present a case of 22 cm large intramuscular mass, located in the psoas muscle of the retroperitoneum. No macroscopic fat was identified on imaging, and the lesion featured extensive myxoid stroma, closely mimicking an intramuscular myxoma on both radiological and histopathological evaluation from biopsy sample. Final diagnosis was however revised to MDM2-negative WDLPS.

This case highlights the diagnostic challenge posed by WDLPS with extensive myxoid stroma lacking MDM2 amplification and macroscopic fat on imaging. Accurate diagnosis relies on the integration of clinical, radiologic, and pathologic data, as well as thorough sampling of the tumor.

## Introduction

Well-differentiated liposarcoma (WDLPS) is the most common subtype of liposarcoma, accounting for 40%-50% of cases [1,2]. It typically affects middle-aged to older adults, with no significant gender predilection, and is most commonly located in the deep soft tissues of the extremities, retroperitoneum, and paratesticular region [1,2]. On CT or MRI, WDLPS typically appears as a well-defined mass composed of both fatty and soft tissue elements. Common radiologic features include thickened septa, nodular nonfatty components, and mild postcontrast enhancement [3,4]. Unlike benign lipomas, WDLPS often demonstrates internal complexity and irregularity within the fatty tissue [3,4].

Histologically, WDLPS is characterized by mature adipose tissue interspersed with broad fibrous septa containing atypical, hyperchromatic stromal cells. While it has a strong tendency for local recurrence, WDLPS generally lacks metastatic potential [1,2]. It is typically associated with amplification of the MDM2 gene, located on chromosome 12q13–15. Immunohistochemistry (IHC) for MDM2 and CDK4 can support the diagnosis, but fluorescence in situ hybridization [5,6] for MDM2 amplification remains the gold standard due to its superior sensitivity and specificity. Molecular testing is particularly important in morphologically ambiguous cases and in distinguishing WDLPS from histologic mimics such as lipoma, myelolipoma, and other soft tissue tumors [[5], [6], [7], [8]].

Occasionally, WDLPS may contain prominent myxoid stroma with cystic degeneration [1,9]. These findings, especially in core biopsy specimens, can mimic other low-grade myxoid neoplasms such as intramuscular myxoma, low-grade myxofibrosarcoma, and low-grade fibromyxoid sarcoma [1,9,10]. In such cases, FISH for MDM2 amplification is critical for accurate diagnosis [11]. However, rare cases of WDLPS may lack MDM2 amplification, posing a diagnostic challenge [[6], [7], [8]].

Herein, we report a diagnostically challenging case of retroperitoneal/psoas muscle WDLPS with extensive myxoid stroma, absence of macroscopic fat, and negative MDM2 gene amplification, which closely mimicked an intramuscular myxoma on both imaging and biopsy.

## Case presentation

A 51-year-old man presented with a large, nontender mass in the left abdomen. He had first noticed the mass while lying down 2 months prior to seeking medical attention. He was otherwise asymptomatic, denying fever, night sweats, weight loss, or changes in bowel or bladder function. His medical history was significant for hypertension, managed with amlodipine. There was no family history of malignancy.

**Fig. 1:**
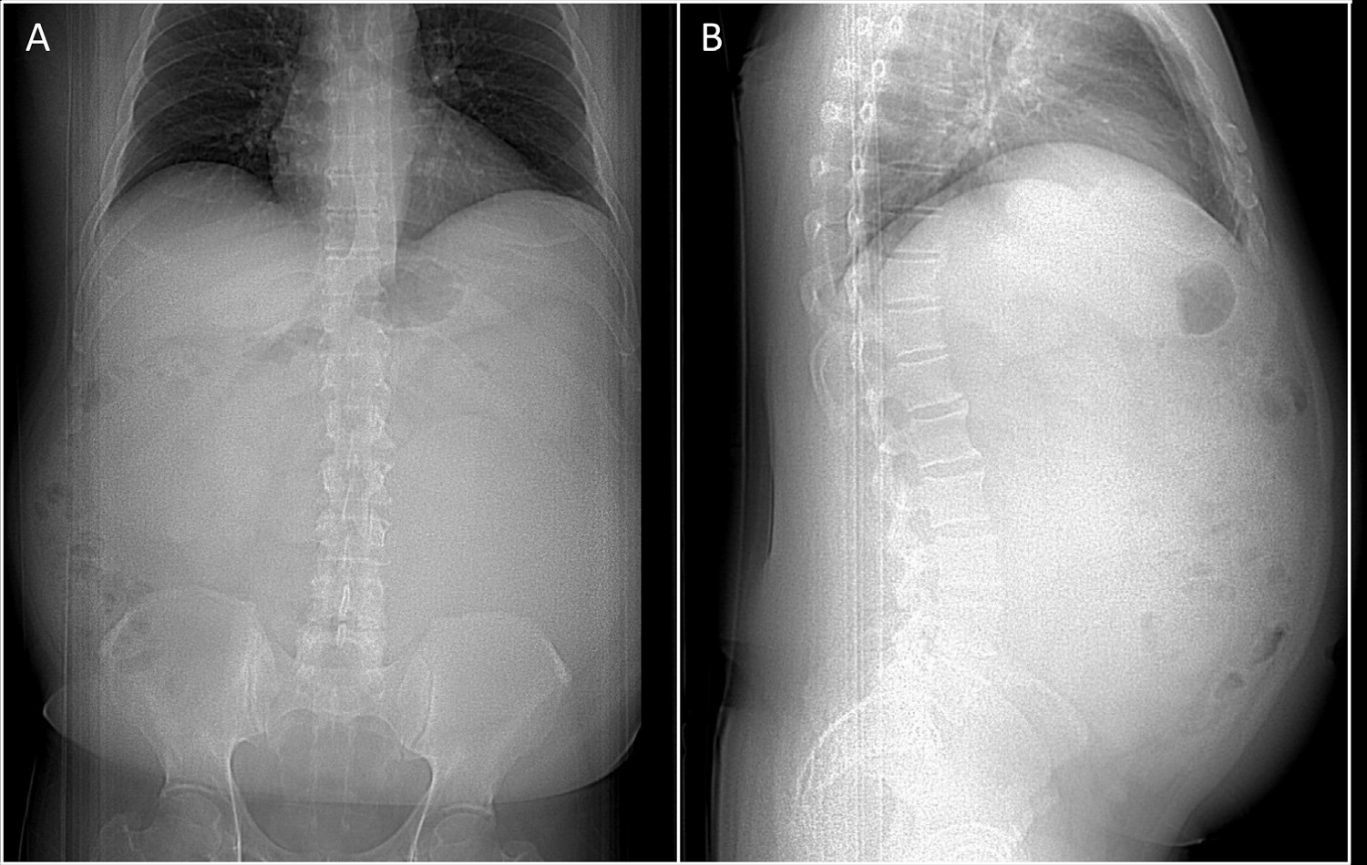
Scout coronal (A) and sagittal (B) CT images of the abdomen and pelvis demonstrate effacement of the iliopsoas muscle shadow and a paucity of bowel gas, suggestive of a potential abdominal mass.

**Fig. 2:**
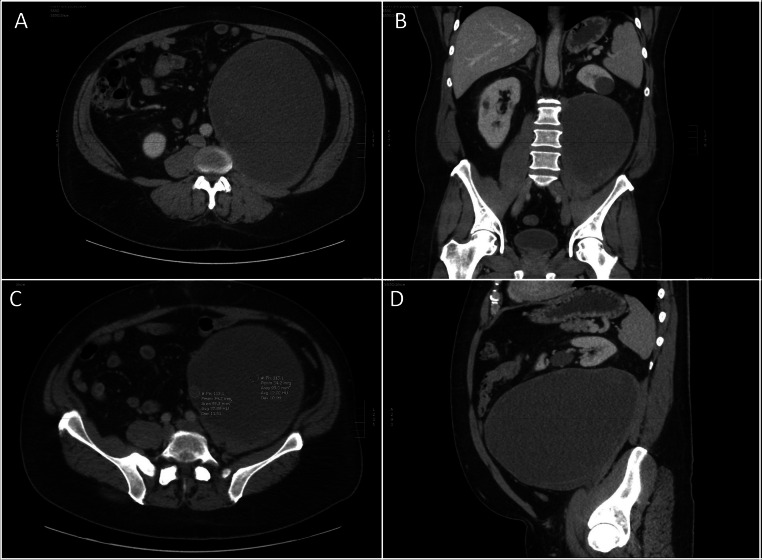
Axial (A) and coronal (B) CT images of the abdomen and pelvis reveal a large, predominantly low-attenuation mass arising from the left iliopsoas muscle, exhibiting a “claw sign.” The mass appears well-circumscribed and extends into the rectoperineal space. (C) The lesion demonstrates central water-like density with peripheral soft tissue attenuation. (D) Sagittal CT image shows superior displacement of the left kidney by the mass. No macroscopic fat component.

On physical examination, a large mass was palpated in the left lower quadrant, extending to but not crossing the midline. Contrast-enhanced CT imaging of the abdomen and pelvis revealed a 22 × 21 × 16 cm, well-circumscribed mass arising from within the left psoas muscle, with a “claw sign,” extending into the retroperitoneal space. The mass displaced the left kidney anteriorly and superiorly but showed no evidence of invasion into adjacent structures outside the psoas. It appeared predominantly cystic, containing multiple septations and peripheral solid components (Fig. 1, Fig. 2). These features raised suspicion for a primary tumor of the psoas muscle.

**Fig. 3:**
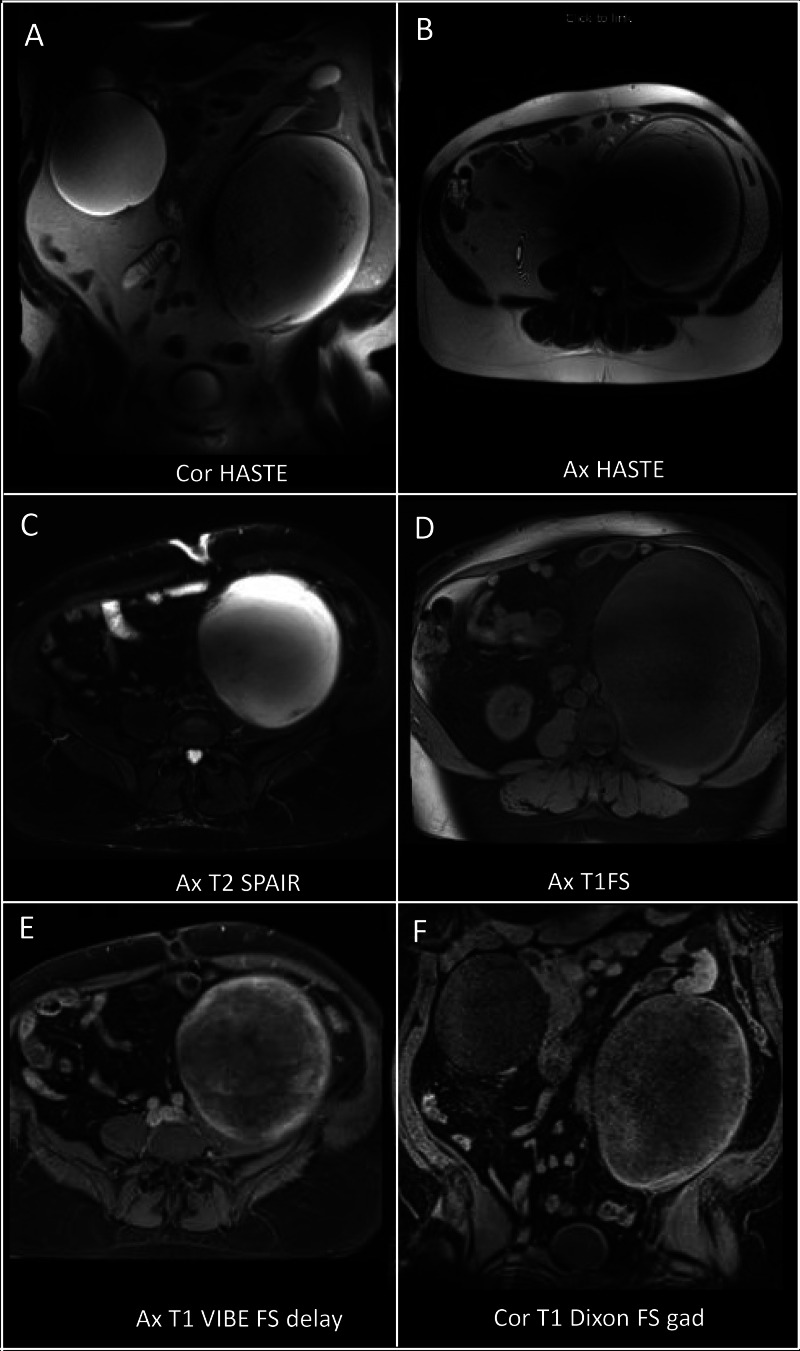
Multisequential and multiplanar MRI of the abdomen and pelvis shows a predominantly T2 hyperintense and T1 hypointense lesion (A–D). The peripheral aspect of the lesion exhibits mild enhancement (E, F). No perilesional edema is noted. No macroscopic fat component.

Subsequent MRI confirmed a large, well-defined mass centered in the left psoas muscle. It exhibited diffuse T2 hyperintensity and T1 hypointensity, with T2 hypointense linear strands throughout. No significant areas of signal drop were observed on T2 fat-saturated sequences to suggest macroscopic fat, and no microscopic fat was detected on chemical shift imaging (data not shown). Postcontrast images demonstrated heterogeneous internal enhancement, predominantly peripheral (Fig. 3). Based on these findings, a myxoid neoplasm versus sarcoma was suspected, and a percutaneous biopsy was recommended.

**Fig. 4:**
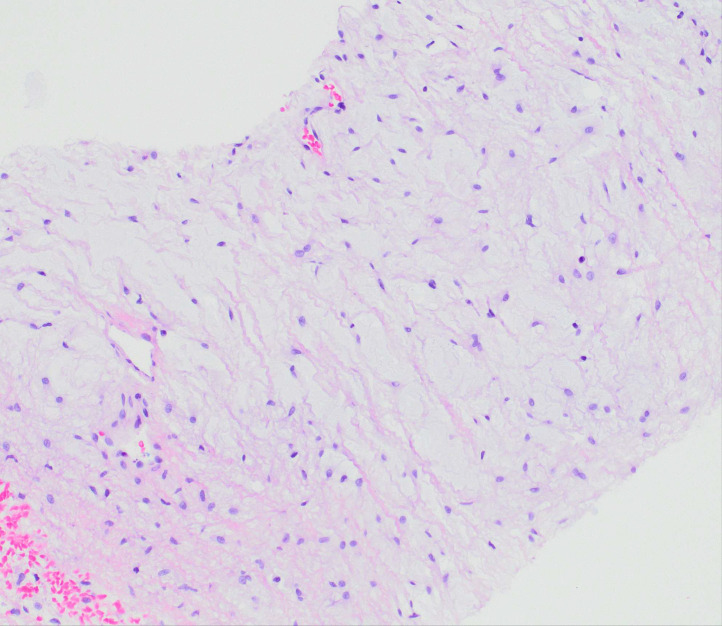
Biopsy reveals a hypocellular neoplasm composed of bland stellate to spindled cells within a prominent myxoid stroma and rare small blood vessels (× 100).

A CT-guided core needle biopsy was performed. Histologically, the specimen showed a hypocellular neoplasm composed of bland stellate to spindle-shaped cells within a prominent myxoid stroma. Scattered small blood vessels were present, but no curvilinear, arcade, or “chicken-wire” vasculature was identified (Fig. 4). There was no significant cytologic atypia, mitotic activity, or tumor necrosis. Immunohistochemically, the tumor cells were positive for CD34 and negative for smooth muscle actin, desmin, SOX10, S100, EMA, MUC4, and STAT6. Fluorescence in situ hybridization [5] for *MDM2* amplification was negative. Based on the combined radiologic and histologic findings, a diagnosis of a low-grade myxoid neoplasm, favoring intramuscular myxoma, wasrendered.

**Fig. 5:**
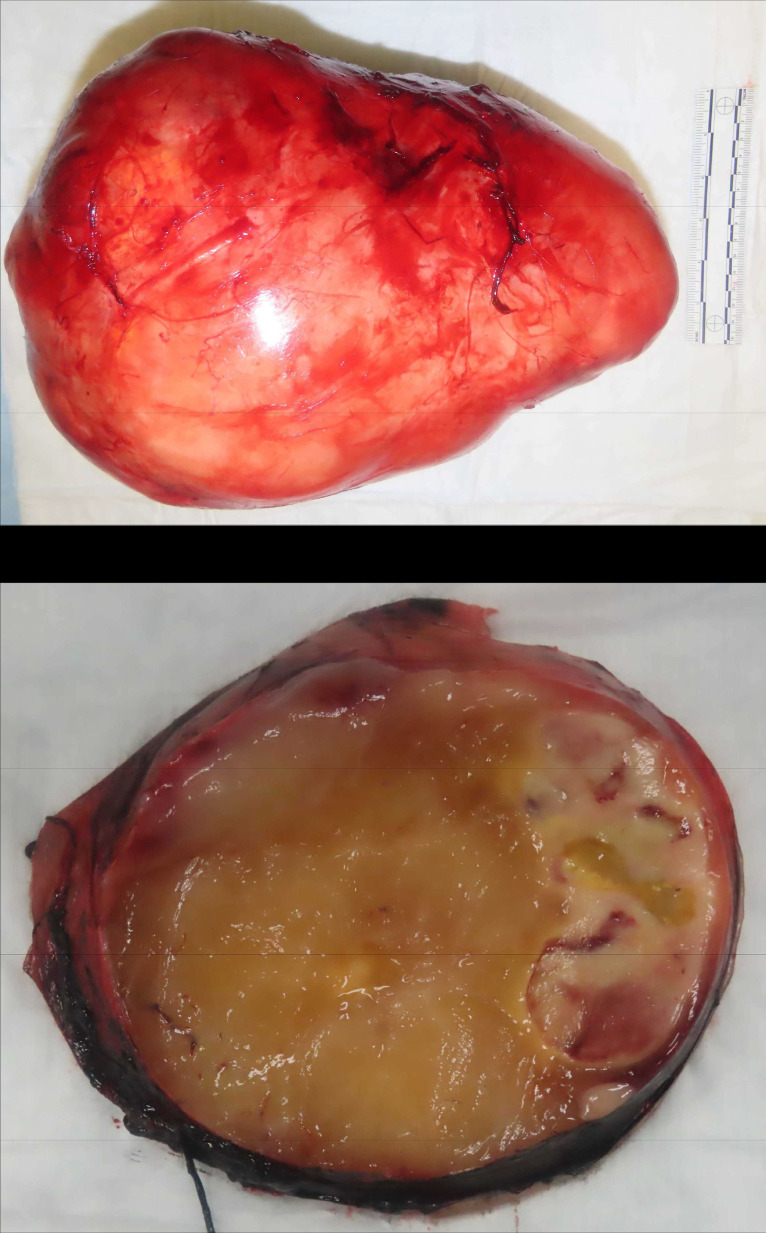
(A) The marginal resection specimen demonstrates a well-circumscribed, encapsulated mass measuring 30 × 25 × 16 cm. (B) The cut surface is predominantly yellow and glistening. Portions of the tumor exhibit a variegated appearance, ranging from white-tan to reddish-yellow, with evidence of cystic degeneration.

**Fig. 6:**
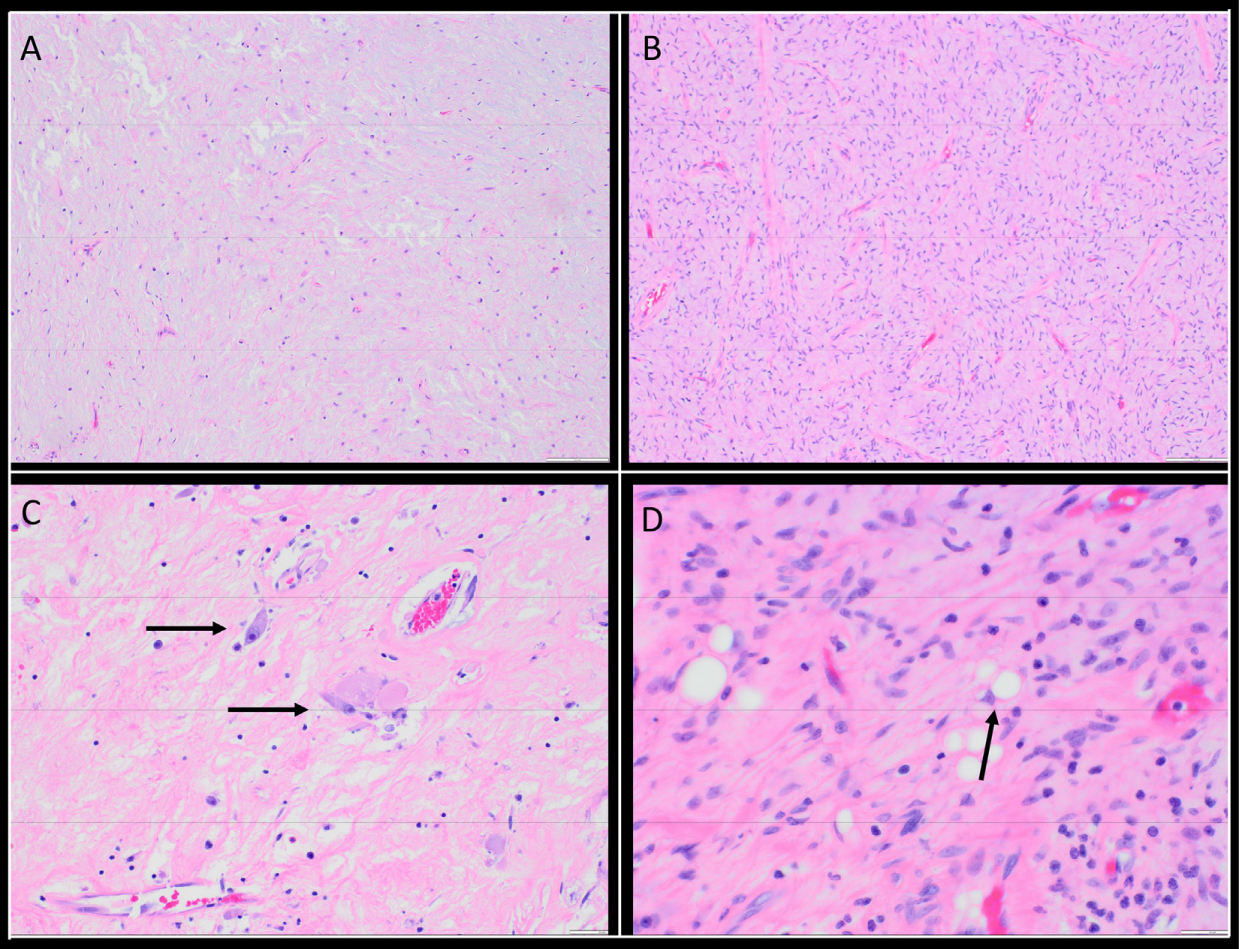
(A) A section from the glistening area of the resection specimen shows features identical to the biopsy (× 100). (B) Low-power view highlights a more cellular area with prominent curvilinear vessels (× 100). (C) Characteristic atypical cells display eosinophilic cytoplasm, enlarged nuclei, and prominent nucleoli (× 200). (D) Rare lipoblasts with multivacuolated cytoplasm and compressed nuclei are also observed (× 400).

The patient subsequently underwent surgical resection. The excised mass was well-circumscribed and encapsulated (Fig. 5), weighing 6267 grams and measuring 30 × 25 × 16 cm. On gross examination, the cut surface was predominantly yellow and glistening (Fig. 6). Approximately 15% of the tumor showed a variegated appearance, ranging from white-tan to reddish-yellow, with areas of cystic degeneration. Scattered regions suggestive of necrosis were also noted.

Histologically, most sections resembled the initial biopsy, displaying hypocellular, hypovascular areas composed of bland spindle to stellate cells in a myxoid stroma (Fig. 6A). However, other areas showed concerning features, including increased cellularity with prominent curvilinear vasculature (Fig. 6B), occasional atypical cells with abundant eosinophilic cytoplasm, enlarged nuclei, and prominent nucleoli (Fig. 6C), as well as rare lipoblasts (Fig. 6D). No definitive mitotic figures or tumor necrosis were identified. Repeat immunohistochemical staining revealed tumor cells positive for CD34 and p16, and negative for smooth muscle actin, desmin, S100, and SOX10, except for the lipoblasts, which were positive for S100 and SOX10.

Based on the tumor’s retroperitoneal location, the presence of irregular fibrous septa containing enlarged, hyperchromatic stromal cells, and the identification of scattered lipoblasts, the final diagnosis was revised to well-differentiated liposarcoma (WDLPS) with myxoid and cystic changes.

## Discussion

We present a diagnostically challenging case of well-differentiated liposarcoma (WDLPS) with extensive myxoid and cystic changes, involving the intramuscular psoas of the retroperitoneum. The tumor mimicked an intramuscular myxoma both radiologically and histologically, and was initially misdiagnosed based on core biopsy findings.

Initial imaging revealed a well-circumscribed, 22 cm mass arising from within the left psoas muscle. On CT, the lesion demonstrated high water content with mild peripheral enhancement. MRI showed uniform low signal intensity on T1-weighted images and high signal intensity on T2-weighted images—features characteristic of myxoid tumors. However, classic MRI features of intramuscular myxoma—such as a thin rim of fat due to adjacent muscle atrophy or perilesional edema—were absent [[12], [13], [14]]. The large size also argued against a myxoma, which typically measures < 5 cm and is most often found in the thigh or buttock [[10], [15], [16]]. Very few cases have been reported in the psoas muscle [12].

Despite these atypical features, the initial core needle biopsy showed a hypocellular, hypovascular neoplasm composed of bland stellate to spindle-shaped cells embedded in a prominent myxoid stroma. There was no cytologic atypia, mitotic activity, or necrosis—features consistent with intramuscular myxoma. Immunohistochemistry showed positivity for CD34 and negativity for smooth muscle actin (SMA), although SMA may be focally positive in some myxomas [10]. FISH analysis for MDM2 gene amplification was negative. Based on radiologic-pathologic correlation—reviewed independently by radiologists at 2 separate institutions—the lesion was diagnosed as a low-grade myxoid neoplasm, favoring intramuscular myxoma.

Following surgical resection, the gross and histologic features raised significant concern for a diagnosis beyond myxoma. Although the cut surface was predominantly gelatinous and resembled a benign myxoma, focal areas displayed variegated, white-tan to reddish-yellow appearance, along with necrosis [2]. Microscopic examination of extensively sampled tissue revealed irregular fibrous septa, occasional atypical hyperchromatic stromal cells, and rare lipoblasts—hallmark features of WDLPS. Despite repeated FISH testing for MDM2 gene amplification on the resection specimen, the result remained negative. Nonetheless, the tumor's retroperitoneal location and the presence of classic histologic features, though focal, were sufficient to support a revised diagnosis of well-differentiated liposarcoma (WDLPS).

### Diagnostic challenges of myxoid WDLPS

While conventional WDLPS is typically straightforward to differentiate from intramuscular myxoma based on clinical, imaging, and histologic features, diagnostic challenges arise when WDLPS undergoes extensive myxoid degeneration [1,9,11]. In these cases, myxoid areas may obscure lipomatous component and mimic benign myxoid neoplasms.

In our case, the extensive myxoid stroma, absence of fat, and hypocellularity created a strong morphologic mimic of intramuscular myxoma on core biopsy. Retrospectively, the large size and retroperitoneal location were atypical for myxoma and should have prompted consideration of a low-grade myxoid malignancy, such as WDLPS or myxoid liposarcoma [10,13]. The negative MDM2 result on biopsy further confounded the diagnosis.

### Overlapping features and differential diagnosis

Both intramuscular myxoma and WDLPS with prominent myxoid stroma belong to the broader group of myxoid neoplasms, which range from benign to high-grade malignant tumors [10]. These tumors share abundant glycosaminoglycan-rich stroma, contributing to similar imaging and histologic features. The differential diagnosis includes: (1) Low-grade fibromyxoid sarcoma (LGFMS): Alternating fibrous and myxoid zones with arcade-like vasculature; positive for MUC4 [10]. (2) Low-grade myxofibrosarcoma: Often hypocellular but with atypical cells and curvilinear vasculature [1,10]. (3) Myxoid liposarcoma: Characterized by round-to-oval cells and prominent chicken-wire vasculature [1,10]. (4) Peripheral nerve sheath tumors: Includes neurofibroma and perineurioma; identified with S100, EMA, GLUT1, and Claudin-1 immunostains.

### Molecular diagnostics and limitations

MDM2 gene amplification is considered the gold standard for diagnosing WDLPS, with a reported sensitivity of 90%-95% [7,8]. Immunohistochemistry for MDM2, CDK4, and p16 can aid in diagnosis, but are less sensitive and specific than molecular testing [[5], [6], [7], [8]]. In rare instances, WDLPS may lack MDM2 amplification, or false-negative FISH results may occur due to limited sampling or low tumor cell content in core biopsies.

In our case, MDM2 amplification was negative on both the initial biopsy and resection specimen. Possible explanations include a false negative, technical failure, or a rare MDM2-negative WDLPS. Although CDK4 amplification can be present in some MDM2-negative cases, it was not assessed in our patient due to unavailability.

## Conclusion

This case highlights a rare presentation of MDM2-negative WDLPS with extensive myxoid stroma, masquerading as an intramuscular myxoma both radiologically and histologically. It underscores the diagnostic difficulty posed by low-grade myxoid neoplasms, particularly in limited biopsy specimens. A multidisciplinary approach integrating clinical, radiologic, histopathologic, and molecular data is essential for accurate diagnosis. Furthermore, this case emphasizes the importance of thorough histologic sampling and gross examination, especially when molecular results are inconclusive.

Although rare, WDLPS without MDM2 amplification remains a recognized diagnostic entity. In such cases, diagnosis must rely on a constellation of findings, including tumor location, size, growth behavior, and characteristic histologic features.

## Ethics approval and consent to participate

Ethics approval and consent to participate was waived because of the retrospective nature.

## Author contributions

All authors have made substantial contributions to all 4 categories established by the International Committee of Medical Journal Editors (http://www.icmje.org) including: (1) conception and design, (2) drafting the article or revising it critically for important intellectual content, (3) final approval of the version to be published.

## Data availability statement

Image data were extracted from the clinical PACS system stored in DICOM standard format. The original data is available upon request.

## Patient consent

Written informed consent was obtained from the patient for publication of this case report and accompanying images. A copy of the written consent is available for review by the Editor-in-Chief of this journal.
